# Efficiency Enhancement of Cocktail Dye of* Ixora coccinea* and* Tradescantia spathacea* in DSSC

**DOI:** 10.1155/2015/582091

**Published:** 2015-12-17

**Authors:** Zularif Zolkepli, Andery Lim, Piyasiri Ekanayake, Kushan Tennakoon

**Affiliations:** ^1^Environmental and Life Sciences Programme, Universiti Brunei Darussalam, Jalan Tungku Link, Gadong BE1410, Brunei Darussalam; ^2^Physical and Geological Sciences Programme, Universiti Brunei Darussalam, Jalan Tungku Link, Gadong BE1410, Brunei Darussalam; ^3^Centre for Advanced Material and Energy Sciences (CAMES), Universiti Brunei Darussalam, Jalan Tungku Link, Gadong BE1410, Brunei Darussalam; ^4^Institute for Biodiversity and Environmental Research (IBER), Universiti Brunei Darussalam, Jalan Tungku Link, Gadong BE1410, Brunei Darussalam

## Abstract

The use of anthocyanin dyes extracted from epidermal leaves of* Tradescantia spathacea* (Trant) and petals of* Ixora coccinea* (IX) was evaluated in the application of dye-sensitized solar cells (DSSCs). Subsequently, cocktail anthocyanin dyes from these dyes were prepared and how they enhanced the cell's overall performance was assessed using five different volume-to-volume ratios. Cocktail dyes absorbed a wider range of light in the visible region, thus increasing the cell efficiencies of the cocktail dyes when compared to the DSSC sensitized by individual dyes. The surface charge (zeta-potential), average size of aggregated anthocyanin molecules (zetasizer), and anthocyanin stability in different storage temperatures were analyzed and recorded. Lower size of aggregated dye molecules as revealed from the cocktail dyes ensured better adsorption onto the TiO_2_ film.* Tradescantia*/*Ixora* pigments mixed in 1 : 4 ratio showed the highest cell efficiency of *η* = 0.80%, under the irradiance of 100 mW cm^−2^, with a short-circuit current density 4.185 mA/cm^2^, open-circuit voltage of 0.346 V, and fill factor of 0.499. It was found that the desired storage temperature for these cocktail dyes to be stable over time was −20°C, in which the anthocyanin half-life was about approximately 1727 days.

## 1. Introduction

Dye-sensitized solar cell (DSSC) is a photovoltaic technology developed by O'Regan and Grätzel in 1991 [1], also known as Grätzel cell. DSSC is designed based on light harvesting by a sensitizing dye attached to the nanostructured semiconductor [1]. Using inorganic dyes as sensitizers had shown great potential for DSSC [1–3]. Study of DSSC using ruthenium-based complex as dye sensitizer [4] and Zn-porphyrin (dye sensitizer) with a Co-based electrolyte [5] achieved maximum efficiencies of 11.5% and 12.3%, respectively.

However, due to the presence of heavy metal complexes, it poses an issue for the environment [6]. This accounts for the use of natural dyes as sensitizers in DSSC. Anthocyanin, chlorophyll, tannin, and carotene, extracted from various plants, fruits, flowers, and leaves, have been successfully used as sensitizers in DSSCs [2, 3]. However, natural dyes as sensitizers showed quite poor performance and instabilities in DSSC. One approach in optimizing the efficiency of DSSCs is by making cocktail dye (mixing two or more dyes) to increase the absorption spectrum of the dyes [7, 8]. Mixed dyes, containing anthocyanin and betalains, had shown overall solar energy conversion efficiency of around 2% [8]. Natural dyes are relatively easy to obtain and extract from plants, reducing the cost manufacturing of DSSC, as opposed to the production of synthetic dyes [1]. Although the currently most efficient Grätzel cell is still less efficient than the standard conventional solar cell, due to the light absorption mechanism of the natural dyes, DSSCs function even in low-light conditions [9].

Anthocyanins are natural components that are responsible for the red-purple color of fruits, flowers, and leaves of plants. They may also present in other plant tissues such as roots, tubers, and stems [2, 3]. They absorb light in the range of 520–550 nm wavelength [10] and are pH dependent, usually red in color in an acidic medium but turning into blue in less acidic condition [6]. Several factors such as light, temperature, and pH can destabilize the anthocyanin molecular structure [11]. Zeta-potential (surface charge), sizes of aggregated dye molecules, pH, and conductivity can be used to determine the stability of anthocyanin [12].

In this study, the performance of a common group of natural dyes abundant in flowers and fruits, namely, anthocyanin, is evaluated in DSSCs. The natural dyes used in this study were obtained from petals of dark red colored* Ixora* sp. (coded as “IX”) and purple lower epidermal leaves of* Tradescantia *sp. (coded as “Trant”).

*Ixora coccinea *(Rubiaceae), also known as flame of wood, is usually grown as ornamental. It is a shrub notable for its bright colored flowers, which are composed of many small blooms massed together into dense, flat-topped flower heads. The flowers contain anthocyanin and have antidiarrheal [13] and cytotoxic properties [14].

*Tradescantia spathacea* is an herbaceous plant that is commercially grown for bedding and rock garden*. T. spathacea* is a plant that adapts to low-light environment and can grow in both shade and exposed light environment. It has anti-inflammatory and anticancer properties [15].

This paper describes the use of these two natural anthocyanin dyes from both plants as cocktail dye in DSSC. Cocktail dye is made by mixing two or more natural dyes together, with the aim of improving the range of absorption of light in the visible region and thus of improving the overall efficiency [16]. The effect of different volume-to-volume ratio concentrations on the photoelectric conversion efficiency of dye-sensitized solar cells is determined.

## 2. Experimental Section

### 2.1. Plant Materials

Petals of* I. coccinea* and lower epidermal leaves of* T. spathacea* were harvested to extract natural anthocyanin dyes.

### 2.2. Anthocyanin Extraction

25 g of petals of* I. coccinea* and lower epidermal leaves of* T. spathacea* was ground with 50 mL of 70% ethanol and stored overnight in the refrigerator at 4°C. On the following day, the extracted samples were stirred using magnetic stirrer for two hours. The procedure continued with the filtration of the samples to remove large residue. Subsequently, the extracts were centrifuged at 4500 rpm using a Denley BS400 (UK) centrifuge machine for five minutes to separate any remaining residues. The next step was to purify the sample using petroleum ether to separate polar and nonpolar components of the extracted dyes.

For* T. spathacea,* additional steps were incorporated after the grinding and purification processes. The extract was placed in a 45–50°C water bath after grinding to dissolve more pigments into the extracting solvent [17] and then kept in a refrigerator at 4°C. During the purification step, petroleum ether was first used and then diethyl ether to remove remaining impurities because it is more nonpolar than petroleum ether. The purified samples were then stored in a reagent bottle at 4°C for further study.

### 2.3. Cocktail Mixtures

The anthocyanin dyes extracted from* T. spathacea* (Trant) and* I. coccinea* (IX) were mixed at different v/v ratios to make five cocktail dye mixtures. For example, 1 mL of Trant was mixed with 4 mL of IX to make a cocktail dye 1 : 4 (v/v). The same method was used to prepare Trant/IX cocktail dyes of ratios 2 : 3 (v/v), 1 : 1 (v/v), 3 : 2 (v/v), and 4 : 1 (v/v).

### 2.4. Anthocyanin Content Determination

The content of anthocyanin presence in the dye extract was determined using the formula described by Giusti and Wrolstad [18] based on the known fact that anthocyanin is pH dependent. Two dilutions of each cocktail dye were prepared before absorbance reading, one with potassium chloride buffer (0.025 M, pH 1.0) and the other with sodium acetate buffer (0.4 M, pH 4.5). The preparation of the reagents was described elsewhere [19]. The absorbance was read at 520 nm and 700 nm against a blank cell containing 70% of ethanol using UV-Vis spectrophotometer (Shimadzu UV-1800, Japan). The monomeric anthocyanin pigment content in the original sample was calculated according to the following formula:

(1) anthocyanin  pigment  content=A×MW×DF×103ε×l,

where *A* = (*A*_520 nm_ − *A*_700 nm_) at pH 1.0 − (*A*_520 nm_ − *A*_700 nm_) at pH 4.5, MW (Molecular Weight) is 449.2 g/mol for cyaniding-3-glucoside, DF is dilution factor, 10^3^ is factor for conversion from g to mg, *ε* is 26900 L mol^−1^ cm^−1^, and *l* is the assumed path length of incident light in cm.

### 2.5. Anthocyanin Absorption Spectrum

Presence of anthocyanin pigments was determined by measuring their absorbance spectra using UV-Vis spectrophotometer (Shimadzu UV-1800, Japan). Pretreated dye extracts with and without concentrated HCl acid were examined in the characterization. Each measurement was repeated thrice to confirm the accuracy of readings.

### 2.6. Zeta-Potential and Zetasizer

The zeta-potential and zetasizer of the individual dyes and cocktail mixtures were measured using Malvern Zetasizer (Nano MAN 0317) at a temperature of 25°C in 70% of ethanol solution. Each measurement was repeated thrice to confirm the accuracy of readings.

### 2.7. Dye-Sensitized Solar Cell Preparation and *I*-*V* Characterization

**Figure 1:**
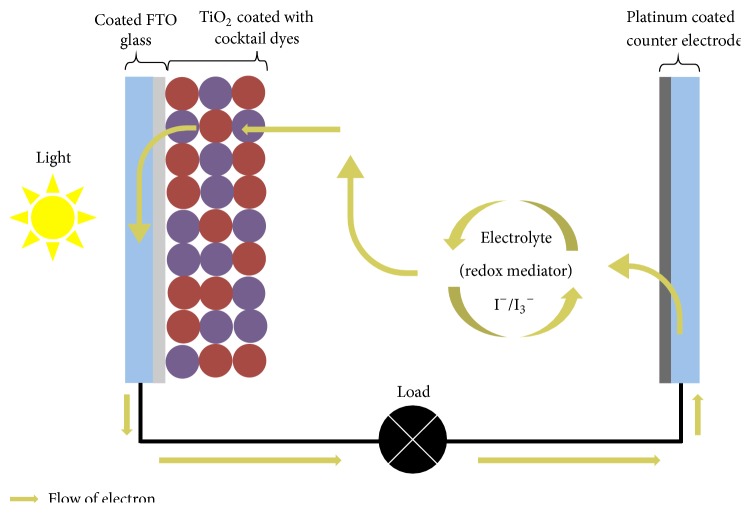
The absorption spectra of the extracts of* Tradescantia spathacea* (Trant) and* Ixora coccinea* (IX) in original and acidified extract.

A conductive glass (FTO) containing sintered TiO_2_ was prepared as the photoanode. The photoelectrodes (anodes) were fabricated using a TiO_2_ paste Solaronix (nanoxide-T, colloidal anatase particles size: ~13 nm, ~120 m^2^ g^−1^ (BET), Switzerland). The TiO_2_ was coated on precleaned fluorine-doped conducting tin oxide (FTO) glasses (Nippon Sheet Glass ~7 Ω sq^−1^) by doctor blade method. Electrodes were preheated (~50°C) using a hair dryer and sintered at 450°C for 30 minutes. The thickness of the TiO_2_ electrodes used for this investigation was ~9 *μ*m (Scanning Electron Microscope, SEM) [20]. The anodes were dipped in respective dye solutions (Trant, IX, and the cocktail dyes) as the photosensitizers. These were left submerged approximately for 16 hours at room temperature in the dark to avoid light exposure. This allows enough time for the dyes to adsorb onto the TiO_2_ porous layer. The anodes were then rinsed with ethanol and air dried. Electrolyte, containing tetrabutylammonium iodide (TBAI, 0.5 M)/*I*_2_ (0.05 M), in a mixture of acetonitrile and ethylene carbonate (6 : 4, v/v), was then introduced between the dyed TiO_2_ electrode and platinum counter electrode [21]. These DSSCs were placed under irradiation of light (100 mW/cm^2^) using sun simulator (DYESOL) for about 4 hours for better incorporation of electrolyte into the TiO_2_ layer [22].  Figure 1 shows a schematic diagram of a functioning DSSC. The performance of the cell in terms of energy conversion efficiency (*η*) was evaluated by using relation of short-circuit current (*I*_sc_), open-circuit voltage (*V*_oc_), and fill factor (FF) as shown below:

(2) $$\begin{array}{c} \left(\;\eta \;\right)=FF\times I_{sc}\times \frac{V_{oc}}{P}, \end{array}$$

where *I*_sc_ is the short-circuit photocurrent density (A cm^−2^), *V*_oc_ is the open-circuit voltage (*V*), *P* is the intensity of the incident light (W cm^−2^), and FF is the fill factor defined as FF = *I*_*m*_ *V*_*m*_/*I*_sc_ *V*_oc_, in which *I*_*m*_ and *V*_*m*_ are the optimum photocurrent and voltage that can be extracted from the maximum power calculated from the *I*-*V* data [20].

### 2.8. Effect of Storage Temperature on Anthocyanin Degradation

A cocktail dye mixture that showed higher conversion efficiency was selected for further evaluation on the impacts of varying temperature regimes for anthocyanin storage. The extracts were stored in enclosed glass bottles to avoid exposure to light at three different storage temperatures −20°C, 4°C, and 25°C. In order to determine the anthocyanin contents, the spectroscopic absorbance of the extracts was initially determined for three consecutive days followed by weekly measurements over a period of one month followed by a final measurement after a 2-month period.

Kinetic parameters of anthocyanin degradation were calculated using the following formulae [23]:

(3) ln⁡CtC0=−k×t,t1/2=−ln⁡0.5k,

where *C*_0_ is initial anthocyanin content and *C*_*t*_ anthocyanin content after* t*-minute storage at the given temperature.

## 3. Results and Discussion

### 3.1. UV-Vis Characterization

**Figure 2:**
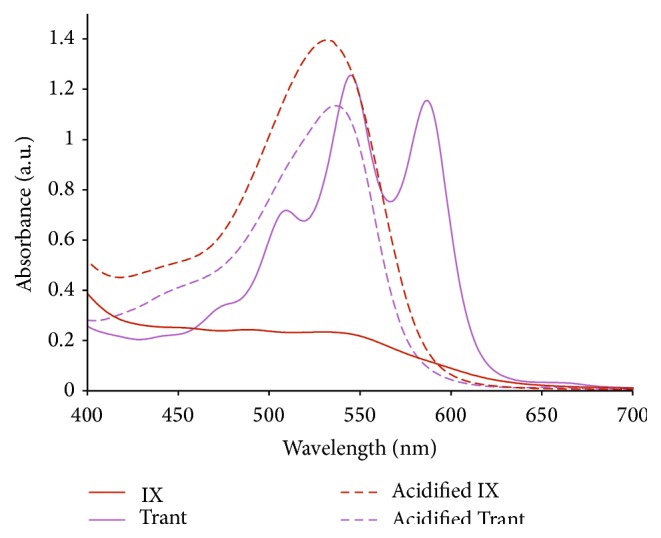
Schematic diagram of a working DSSC.

The presence of anthocyanin dyes was confirmed using UV-visible spectrophotometer, where peaks at 525 nm and 540 nm after acidification of IX and Trant dyes, respectively, indicate the presence of anthocyanidin (see Figure 2) [24]. Acids remove the sugar component, which converts anthocyanin into anthocyanidin that give absorption spectra peaks in the region of 490~570 nm [25]. The three distinct peaks at 511 nm, 548 nm, and 588 nm of the Trant curve suggested that the extracts might as well contain chlorophylls, which is absorbed most strongly in the blue and red regions of the absorption spectra.

**Figure 3:**
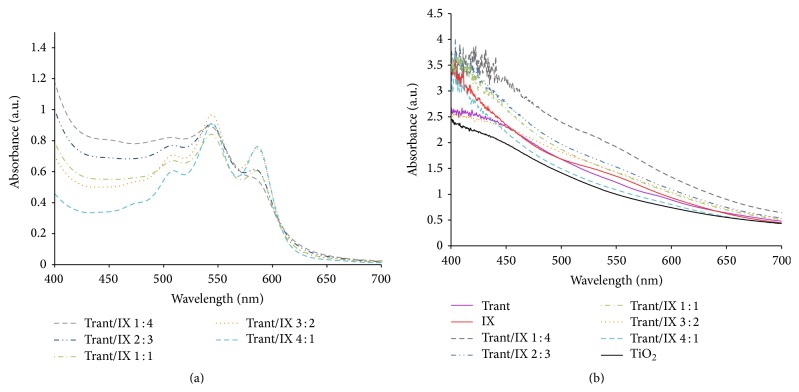
The absorption spectra of cocktail dyes* Tradescantia spathacea* (Trant) and* Ixora coccinea* (IX) (a) before and (b) after adsorption onto TiO_2_ film.

Figure 3(a) shows the absorption spectra for five cocktail dye mixtures before the adsorption of dyes onto the TiO_2_ paste. The figure shows the maximum absorption peak for all cocktail dye mixtures at 550 nm, and for the cocktail mixture, the absorption range varies within 490~650 nm.  Figure 3(b) shows the absorption spectra for five cocktail dye mixtures after adsorption onto the TiO_2_ paste. Results show that the cocktail dyes after adsorption onto TiO_2_ paste have increased the range of the absorption wavelength and have allowed further absorption of more visible light. This has led to further increase of the photoelectric conversion efficiency of the DSSC. The highest absorbance readings were from cocktail mixture Trant/IX 1 : 4, which contains more IX, while the lowest absorbance readings were from cocktail mixture Trant/IX 4 : 1, which had the lowest concentration of IX among the mixtures. The absorption readings have decreased as the concentrations of Trant to IX increased. This shows that TiO_2_ dyed with IX might have better absorption capability compared to TiO_2_ dyed with Trant, and this can be attributed to its homogeneous adsorption onto the TiO_2_ surface that increases the absorption of light in the visible region [2].

### 3.2. Zeta-Potential and Zetasizer

**Figure 4:**
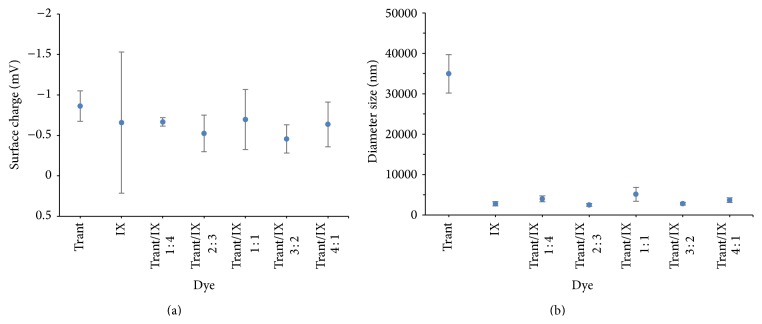
(a) Zeta-potential and (b) zetasizer of individual anthocyanin dyes extracted from* Tradescantia spathacea* (Trant) and* Ixora coccinea* (IX) and cocktail mixtures prepared from different combinations of these pigment extracts.

Experimental results showed that average zeta-potentials of anthocyanin extracted from IX, Trant, and the cocktail mixtures prepared using different ratios of these pigment extracts occurred between −0.4 mV and −0.9 mV (Figure 4(a)). This value indicates a high possibility for all tested anthocyanin dyes undergoing molecular aggregations and showing increased instability of colloids. This is because the least stable colloidal systems have no force to repel molecule that can cause high charge transfer resistance that in turn hinders the electron injection efficiency during the *I*-*V* performance [26, 27]. The aggregation could also result in poor physical contacts between the anthocyanins pigment and the TiO_2_ layer, which causes desorption of dyes from the TiO_2_ layer [28].

As depicted in Figure 4(b), the average size of aggregated dye molecules of Trant is 34 900 nm while IX dyes reached a dimension of 2810 nm. However, all the cocktail dyes had average sizes in the range of 2500 to 5120 nm, approximately 6 to 7 times lower when compared to the individual pigment of anthocyanins extracted from* T. spathacea*. The small aggregated sizes indicate an increase in the surface area and thus, a large amount of the dyes can adsorb onto the TiO_2_. This resulted in the increased absorption by the dyes as shown in the absorption spectra of IX and cocktail mixtures of Figures 3(a) and 3(b) and increased efficiencies of DSSC prepared by cocktail dye mixtures (see Table 1). Hwang et al. have reported that the photocurrent densities increase with higher adsorption of dyes that may in turn give superior characters to DSSCs owing to the low charge transfer resistance from the monolayer adsorption [29].

### 3.3. *I*-*V* Plot

**Figure 5:**
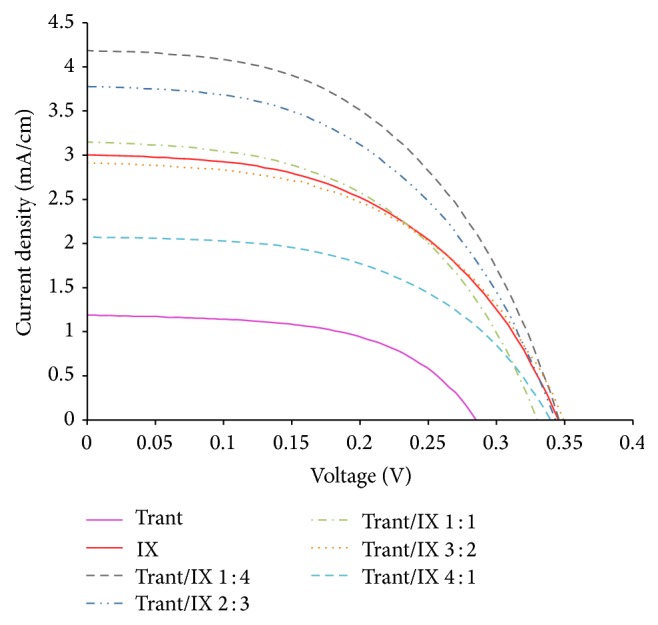
Current-voltage characteristics of the DSSCs sensitized with anthocyanin extracted from* Tradescantia spathacea* leaves (Trant) and* Ixora coccinea* flowers (IX).

Figure 5 and Table 1 show the photoelectrical parameters for a DSSC that is sensitized using five cocktail dye mixtures from anthocyanins pigment extracted from lower epidermis of* T. spathacea *leaves and* I. coccinea* flowers. Compared to the individual dyes, cocktail dyes exhibit reasonable conversion efficiencies. This is attributed to better absorption intensity, and the absorption wavelength range is broader than in those individual dyes' extract only (Figure 3). Similar results were reported by Kimpa et al. [30], who also studied the performance of cocktail dye in DSSC, where sensitized cocktail dye has exhibited better result than individual dye alone. It is also evident that the short-circuit current or the open-circuit voltage of DSSC sensitized by the individual dyes is lower than that sensitized by the cocktail dyes. Therefore, the efficiencies of photoelectric conversion of DSSC sensitized by the cocktail dyes are higher than the individual dye (Trant and IX) constituents.

DSSC sensitized with cocktail dye ratio 1 : 4 (v/v) produces the highest* I-V* performance with *η* = 0.80% with open-circuit voltage (*V*_oc_) of 0.343 V, current density (*J*_sc_) of 4.185 mA/cm^2^, and fill factor of 0.499, under the irradiance of 100 mW cm^−2^. This might be due to the lower concentration of Trant to IX in the cocktail dye ratio 1 : 4 (v/v). IX better performance than Trant in DSSC might be because anthocyanin of* I. coccinea *favors a monolayer adsorption, which optimizes the DSSC efficiency [29, 31], and homogeneous adsorption on the TiO_2_ surface [6].

### 3.4. Photodegradation Kinetics

**Figure 6:**
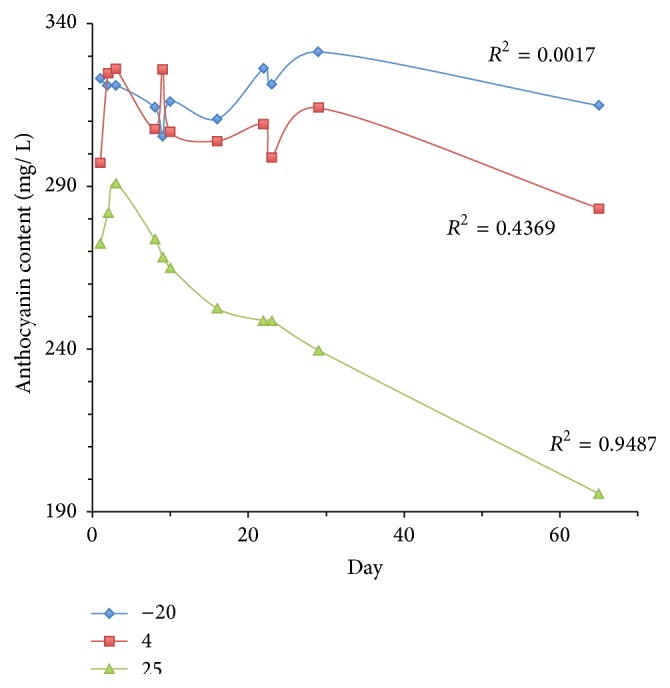
Degradation of anthocyanin pigments of cocktail dye mixture of* Tradescantia spathacea* (Trant) and* Ixora coccinea* (IX) in 1 : 4 ratio at three different storage temperatures (−20°C, 4°C, and 25°C) over a two-month period.

Stability of the anthocyanin pigment in the cocktail dye mixture Trant/IX 1 : 4 was relatively high when they were stored at −20°C and 4°C but decreased progressively when pigments were stored at 25°C over a two-month period (see Table 2 and Figure 6). The cocktail mixture was shown to be most stable when stored at −20°C with a percentage loss of 2.53%, followed by the storage at 4°C (4.72%) and 25°C (28.20%), respectively.

Result showed significantly faster anthocyanin degradation rates when they were stored at 25°C. The degradation rates are represented by half-life (*t*_1/2_) values; the higher the half-life value, the slower the degradation rate. The highest half-life period (1727.05 days) was recorded from anthocyanin pigments which was in the cocktail dye mixture Trant/IX 1 : 4 stored at −20°C. The half-life was reduced to 133.88 days, when it was stored at 25°C over a period of two months.

Previous study of pigments from* Melastoma malabathricum, Hibiscus rosa-sinensis, *and* Codiaeum variegatum* stored at −20°C showed *t*_1/2_ values of 539.13, 336.37, and 405.72 days, respectively. The *t*_1/2_ values of those pigments which were stored at 25°C were markedly reduced to 110.71, 219.74, and 254.25 days, respectively [2]. Kırca and Cemeroğlu also have suggested that the lowest storage temperature should be used where possible to minimize the anthocyanin degradation [32]. This is in line with our observations where the most stable anthocyanin extracts of cocktail dyes have been obtained when they had been stored at −20°C over a period of two months.

## 4. Conclusions

Five cocktail mixtures (different v/v ratios) of dyes extracted from* Ixora coccinea* (IX) flower petals and* Tradescantia spathacea* (Trant) lower epidermal peels were assessed as potential candidates to increase the efficiency in DSSCs. Zeta-potential study revealed that the cocktail dyes have a tendency to agglomerate. The aggregated molecule sizes were determined (between 2500 and 5120 nm) in cocktail dyes. Smaller sized aggregated dye molecules aid in the adsorption of dyes onto the TiO_2_ layer of DSSC. Cocktail dyes exhibited higher conversion efficiencies than the pigments extracted from IX flowers and Trant leaves individually. Thus, this work shows the effectiveness of cocktail sensitizers in DSSCs. Best overall energy conversion efficiency (*η* = 0.80%) was obtained from the DSSC sensitized with the cocktail prepared in the Trant/IX ratio 1 : 4 (v/v). This cocktail dye was most stable when stored at −20°C. Modifications of cocktail dyes as reported here will further enhance the stability of zeta-potential to further increase the affinity of the dyes onto TiO_2_ nanocrystalline thin films. Although the solar-to-electrical energy conversion efficiencies recorded here were lower than those measured for silicon based solar cells, our DSSC developed with a cocktail dye mixture prepared using two tropical plant sources shows promising potential for further improvements in relation to its efficiency, stability, and commercialization.

## Figures and Tables

**Table 1:** The photoelectric parameters of DSSCs sensitized with natural dyes extracted from the lower epidermis of *Tradescantia spathacea* leaves (Trant) and flowers of *Ixora coccinea* (IX).

Dyes	*V*_oc_ (V)	*J*_sc_ (mA/cm^2^)	FF	*η* (%)
Individual dyes				
Trant	0.285	1.189	0.554	0.21 ± 0.02
IX	0.344	3.005	0.507	0.56 ± 0.02
Cocktail dyes (Trant/IX)				
1 : 4	0.346	4.185	0.499	0.80 ± 0.02
2 : 3	0.343	3.780	0.492	0.71 ± 0.02
1 : 1	0.331	3.150	0.502	0.59 ± 0.02
3 : 2	0.359	2.914	0.492	0.61 ± 0.02
4 : 1	0.344	2.074	0.520	0.41 ± 0.02

**Table 2:** Kinetic parameters of anthocyanin degradation in cocktail dye mixture of *Tradescantia spathacea* (Trant) and *Ixora coccinea* (IX) in 1 : 4 ratio at three different storage temperatures (−20°C, 4°C, and 25°C).

Temperature (°C)	Initial conc. (mg/ L)	Final conc. (mg/ L)	Percentage loss (%)	*t*_1/2_ (day)
−20	322.96	314.77	2.53	1727.05
−4	297.24	283.21	4.72	917.48
25	272.36	195.54	28.20	133.88
